# Development of Rice Stripe Tenuivirus Minireplicon Reverse Genetics Systems Suitable for Analyses of Viral Replication and Intercellular Movement

**DOI:** 10.3389/fmicb.2021.655256

**Published:** 2021-03-23

**Authors:** Xiaoyan Zhang, Kai Sun, Yan Liang, Shuo Wang, Kaili Wu, Zhenghe Li

**Affiliations:** ^1^State Key Laboratory of Rice Biology, Institute of Biotechnology, Zhejiang University, Hangzhou, China; ^2^Ministry of Agriculture Key Laboratory of Molecular Biology of Crop Pathogens and Insect, Zhejiang University, Hangzhou, China; ^3^Key Laboratory of Biology of Crop Pathogens and Insects of Zhejiang Province, Zhejiang University, Hangzhou, China

**Keywords:** rice stripe virus, tenuivirus, bunyavirus, minireplicon, reverse genetics, codon optimization

## Abstract

Rice stripe virus (RSV), a tenuivirus with four negative-sense/ambisense genome segments, is one of the most devastating viral pathogens affecting rice production in many Asian countries. Despite extensive research, our understanding of RSV infection cycles and pathogenesis has been severely impaired by the lack of reverse genetics tools. In this study, we have engineered RSV minireplicon (MR)/minigenome cassettes with reporter genes substituted for the viral open reading frames in the negative-sense RNA1 or the ambisense RNA2-4 segments. After delivery to *Nicotiana benthamiana* leaves via agroinfiltration, MR reporter gene expression was detected only when the codon-optimized large viral RNA polymerase protein (L) was coexpressed with the nucleocapsid (N) protein. MR activity was also critically dependent on the coexpressed viral suppressors of RNA silencing, but ectopic expression of the RSV-encoded NS3 silencing suppressor drastically decreased reporter gene expression. We also developed intercellular movement-competent MR systems with the movement protein expressed either *in cis* from an RNA4-based MR or *in trans* from a binary plasmid. Finally, we generated multicomponent replicon systems by expressing the N and L proteins directly from complementary-sense RNA1 and RNA3 derivatives, which enhanced reporter gene expression, permitted autonomous replication and intercellular movement, and reduced the number of plasmids required for delivery. In summary, this work enables reverse genetics analyses of RSV replication, transcription, and cell-to-cell movement and provides a platform for engineering more complex recombinant systems.

## Introduction

Rice stripe virus (RSV) has caused dramatic reductions in rice yield and quality among Asian countries, especially in China, Japan, and Korea (Hibino, 1996; Abo and Sy, 1998; Wang et al., 2008). In nature, RSV is transmitted exclusively by small brown planthoppers (*Laodelphax striatellus* Fallén) in a persistent and circulative-propagative manner (Liu et al., 2018) and infects several monocotyledonous crop species, including rice, maize, wheat, oat, and foxtail millet (Falk and Tsai, 1998). Under laboratory conditions, RSV can also infect the dicotyledonous model plants *Nicotiana benthamiana* (Xiong et al., 2008) after mechanical inoculation and *Arabidopsis thaliana* during viruliferous insect feeding (Sun et al., 2011).

*Rice stripe virus* is the type species of the genus *Tenuivirus* in the family *Phenuiviridae* (Kuhn et al., 2020). The RSV genome consists of four negative-sense, single-stranded RNAs that encode seven proteins (Cho et al., 2013). The 18–20 bases at the 5′ and 3′ terminal sequences of the four RNA segments are conserved and are almost complementary to each other to form panhandle structures that presumably are involved in replication and transcription (Takahashi et al., 1990; Wu et al., 2013). RNA1, the largest genomic RNA segment, contains a large RNA-dependent RNA polymerase (L or RdRp) open reading frame (ORF) in the complementary (c)RNA sequence (Barbier et al., 1992; Toriyama et al., 1994). RNA2, RNA3, and RNA4 are ambisense and each encodes two ORFs separated by a non-coding intergenic region (IGR) (Zhu et al., 1991, 1992; Hamamatsu et al., 1993). The virion-sense (v)RNA2 segment contains an NS2 ORF, and the complementary RNA2 (cRNA2) encodes a glycoprotein precursor (GP) (Takahashi et al., 1993). The exact function of NS2 is not clear, but it may participate in RNA silencing suppression and systemic movement (Du et al., 2011; Zheng et al., 2015), whereas the glycoprotein is involved in insect vector transmission (Lu et al., 2019). vRNA3 encodes a non-structural protein (NS3) that functions as a robust viral suppressor of RNA (VSR) (Xiong et al., 2009; Shen et al., 2010), and cRNA3 encodes a nucleocapsid (N) protein that tightly encapsidates the genome segments along their entire length (Hayano et al., 1990). RSV vRNA4 encodes a disease-specific protein (SP), and cRNA4 encodes a movement protein (MP) (Xiong et al., 2008; Kong et al., 2014).

Reverse genetics technologies allow manipulation of RNA virus genomes through their complementary DNA (cDNA) copies to evaluate the effects of targeted genome alterations on virus biology and pathogenesis. However, application of reverse genetics to negative-stranded RNA viruses (NSVs) like RSV has been a challenge. This is because the NSV genome must be encapsidated by the N protein and the associated L (RdRp) protein to assemble a nucleocapsid or ribonucleoprotein (RNP) complex that can function in replication and transcription. Hence, biologically active RNPs must be reconstituted *in vivo* in order to evaluate the effects of mutations introduced into the recombinant viral genomes (Walpita and Flick, 2005; Jackson and Li, 2016). Owing to the low efficiencies of RNP assembly and additional technical barriers, genetic manipulation of plant-infecting NSVs has been extremely challenging (Jackson and Li, 2016). Fortunately, several technical breakthroughs have recently been made with the rhabdoviruses Sonchus yellow net virus (SYNV) (Wang et al., 2015; Ma and Li, 2020) and barley yellow striate mosaic virus (Gao et al., 2019), tomato spotted wilt tospovirus (TSWV) (Feng et al., 2020a), and rose rosette emaravirus (Verchot et al., 2020) that permit recombinant studies.

Although the general principles of plant NSV recombinant systems are similar, the success of each system lies in tackling specific technical obstacles (Jackson and Li, 2016; Feng et al., 2020b; German et al., 2020; Zang et al., 2020). A prelude to fully infectious virus recoveries is the establishment of a minireplicon (MR)/minigenome system to optimize the conditions needed for optimum RNP reconstitution. MRs are derivatives of viral RNA that contains all the necessary elements for encapsidation, replication, and gene expression, with all or some of the viral ORFs being replaced by reporter genes. MR reporter gene expression provides a facile and trackable means to validate the functionality of viral RNAs and proteins and to devise optimum conditions for fully recombinant virus rescues (Walpita and Flick, 2005; Jackson and Li, 2016). In addition, MR rescue systems *per se* are valuable for reverse genetics dissection of the roles of viral *cis*-acting elements and *trans*-acting factors in replication and transcription and for screening of antiviral chemicals or biological factors (Whelan et al., 2004; Su et al., 2015).

RSV has been the subject of extensive studies owing to its economic importance, and substantial progress has been made in recent years in understanding the intimate virus–plant and virus–insect interactions (Falk and Tsai, 1998; Fu et al., 2018; Huo et al., 2018; Liu et al., 2018; Qin et al., 2018; Lu et al., 2019; Chen et al., 2020; Yang et al., 2020). However, the lack of reverse genetic systems for RSV and for tenuiviruses in general presents a critical technological gap that has severely hampered research on RSV infection cycles and pathogenesis. In this study, we have devised a strategy to facilitate expression of the exceedingly large RSV L protein (about 337 kDa) through codon optimization and to permit successful development of MR systems derived from each of the four RSV genome segments. The utility of the MR systems is demonstrated by reverse genetics analyses of the function of viral *cis*-elements and the NS3 protein in replication. The MR systems also permit studies of the intercellular movement of the *in vivo* reconstituted viral RNPs.

## Materials and Methods

### Plant and Virus Source

The *N. benthamiana* plant was grown in a glasshouse maintained at 25°C with 16 h light/8 h dark cycle under 60% relative humidity. Four- to six-week-old plants were used for agroinfiltration. RSV-infected rice plants were collected from Zhejiang Province, China. The RNA1–RNA4 sequences are available at the GenBank under the accession numbers MW463890, MW463891, MW463892, and MW463893.

### Plasmid Construction

To construct binary plasmids for expression of RSV L, N, NS3, and MP proteins, we amplified their coding sequences from cDNAs reverse transcribed from total RNA extracted from RSV-infected rice plants by using the primer pairs L/*Bam*HI/F and L/*Sal*I/R, N/*Bam*HI/F and N/*Sal*I/R, NS3/*Bam*HI/F and NS3/*Sal*I/R, and MP/*Bam*HI/F and MP/*Sal*I/R (Supplementary Table 1). PCR products were inserted into the *Bam*HI and *Sal*I double-digested pGD binary vector (Goodin et al., 2002) by ClonExpress II One Step Cloning (Vazyme, Nanjing, China). The codon-optimized L code sequence was chemically synthesized in the cloning vector pUC57 by GenScript Biotech Crop (Nanjing, China). The optimized sequence, as provided in Supplementary Figure 1, was released from the cloning vector, and the pCB301-2μ *Escherichia coli*–*Agrobacterium*–yeast shuttle plasmid (Sun et al., 2017) was linearized by PCR with the primers pCB/L-opt/F and pCB/L-opt/R. Mixtures of the two fragments were transformed into yeast cells to obtain the circularized pCB301-2μ-L-opt plasmid using the yeast homologous recombination-based cloning protocol described previously (Sun et al., 2017). Plasmids assembled in yeast cells were purified using TIANprep Yeast Plasmid DNA Kit (Tiangen, China) and transformed into *E. coli* top 10 competent cells for further propagation. To generate the L-opt^dDD^ binary vector, two amino acid deletions were created in the pUC57 cloning vector by site-directed mutagenesis with the primers L-opt/del DD/F and L-opt/del DD/R. Clones with correct deletions were verified by Sanger sequencing, and the full-length L mutant sequence was inserted into the pCB301-2μ shuttle vector as described above. All primers used for cloning are shown in Supplementary Table 1.

To generate the vRNA1 MR_GFP_ transcription plasmid, we amplified the RNA1 cDNA with the vRNA1/pCB/F and vRNA1/pCB/R primers and inserted the cDNA into a *Stu* I and *Sma* I double-digested pCB301-2μ plasmid. Subsequently, this plasmid was linearized by PCR with the vRNA1/backbone/F and vRNA1/backbone/R primers to remove the L coding sequence, and the linearized DNA was ligated with the green fluorescent protein (GFP) fragment amplified with the primers GFP/F and GFP/R by using ClonExpress MultiS One Step Cloning Kit (Vazyme, China).

To generate the transcription plasmid vRNA3_RFP__–__GFP_, RNA3 cDNA with partial HHRz was amplified with the primers vRNA3/HHRzpart/F and vRNA3/pCB/R, and the pCB301 plasmid containing the double 35S promoter and HDV ribozyme (Yao et al., 2011) was linearized by PCR with the 35S/HHRz/R and HDV/F primers. The two fragments were ligated by using the In-Fusion cloning protocol to produce an intermediate plasmid pCB301-vRNA3. Next, the NS3 and N genes in the pCB301-vRNA3 were replaced with red fluorescence protein (RFP) and GFP to generate pCB301-vRNA3 MR_RFP__–__GFP_. This was accomplished by amplifying the GFP, RFP, and IGR3 fragments with the primer pairs GFP/F and GFP/R, RFP/F and RFP/R, and RNA3/IGR/F and RNA3/IGR/R, respectively, and these fragments were inserted into the PCR-linearized pCB301-vRNA3 with the primers vRNA3/backbone/F and vRNA3/backbone/R.

To delete the 5′ or 3′ untranslated regions (UTRs) in vRNA3 MR_RFP__–__GFP_, fragments lacking the 5′ or 3′ UTRs were amplified with the RNA3/del 5′ UTR/F and vRNA3/pCB/R, or the vRNA3/HHRzpart/F and RNA3/del 3′ UTR/R primer pairs, respectively. The resulting fragments were inserted into the pCB301 vectors that were linearized by PCR with the primers 35S/HHRz/R and HDV/F. To eliminate the IGR3 in vRNA3 MR_RFP__–__GFP_, the vRNA3 MR_RFP__–__GFP_ plasmid was used as a template for amplification of two overlapping fragments excluding the IGR3 region, with the vRNA3/HHRzpart/F and RFP/R, GFP/RFP/R and vRNA3/pCB/R primer pairs. These two fragments were then ligated by In-Fusion cloning.

To generate the vRNA2 MR_RFP__–__GFP_ transcription vector, we first amplified the vRNA2 cDNA with the primers vRNA2/HDV/R and vRNA2/HHRz/F and ligated the product with the PCR-linearized pCB301 vector with the HHRz/backbone/F and HDV/R primers. Next, the NS2 and GP genes were replaced by RFP and GFP by inserting the GFP, RFP, and IGR2 fragments into linearized vRNA2_NS__2__–GP_ with the vRNA2/backbone/F and vRNA2/backbone/R primers. To construct the vRNA2 MR_NS__2__–GFP_ plasmid, a fragment containing the NS2 gene and the IGR was amplified from the RNA2 cDNA with the vRNA2/pCB/F and vRNA2 IGR/R primers. A fragment containing the GFP gene and UTRs was also amplified from vRNA2 MR_RFP__–__GFP_ vector with the primers GFP/R and vRNA2/HDV/R. The two fragments were inserted into *Stu* I and *Sma* I doubled-digested pCB301 by In-Fusion cloning.

To produce cRNA1_L–opt_, the cRNA1 cDNA was amplified from infected rice plants with the cRNA1/pCB/F and cRNA1/pCB/R primers and inserted into the *Stu* I and *Sma* I double-digested pCB301-2μ plasmid to generate pCB301-2μ-cRNA1. Next, a linear fragment excluding the L coding region was amplified from this plasmid with the primers cRNA1/backbone/F and cRNA1/backbone/R and mixed with the fragment containing the codon-optimized L coding sequence. The mixtures were transformed into yeast cells to generate the circularized plasmid pCB301-2μ-cRNA1_L–opt_ via yeast homologous recombination.

To generate a plasmid for transcription of cRNA3 MR_N–RFP_, total RNA extracted from RSV-infected rice was used to amplify a fragment spanning the N and IGR3 region with the cRNA3/pCB/F and RNA3/IGR/F primer pair, and a fragment containing RFP was amplified from the vRNA3 MR_RFP__–__GFP_ plasmid with the RFP/R and cRNA3/pCB/R primers. Next, the two fragments were inserted into the *Stu* I and *Sma* I doubled-digested pCB301 by In-Fusion cloning.

To engineer a plasmid for transcription of cRNA4 MR_MP–RFP_, we first amplified the RNA4 cDNA by PCR with the cRNA4/pCB/F and cRNA4/pCB/R primers and inserted the fragment into the *Stu* I and *Sma* I doubled-digested pCB301 to produce the pCB301-cRNA4 plasmid. Thereafter, the SP gene was replaced by RFP by In-Fusion cloning-based ligation of the RFP fragment amplified with the RFP/F and RFP/R primers and the vector fragment amplified with the cRNA4/backbone/F and cRNA4/backbone/R primers.

### Northern Blot

Total RNA extracted from infiltrated leaves was separated in 2% agarose-formaldehyde gels, followed by transfer to Hybond-N + membranes (GE Healthcare, United Kingdom). The membranes were probed with the digoxin-labeled sense (caccatcttcttcaaggacgacggcaactacaagacccgcgccgaggtgaagttcgagg) or antisense (cctcgaacttcacctcggcgcgggtcttgtagttgccgtcgtccttgaagaagatggtg) GFP oligonucleotide probes (Invitrogen, Shanghai, China). The membranes were probed by DIG High Prime DNA Labeling and Detection Starter Kit II following the manufacturer’s instructions (Roche, Basel, Switzerland).

### MR Assays by Agroinfiltration

For RSV MR assays, the binary plasmids described above, as well as the pCB-VSRs plasmid containing the tandem expression cassettes encoding p19, Hc-Pro, and γb (Sun et al., 2017), or the pGD binary plasmids encoding the individual VSRs (Ganesan et al., 2013), were introduced into *Agrobacterium* (EHA105 strain) by electroporation. For SYNV MR assays, the *Agrobacterium* strains containing the binary plasmids for expression of SYNV MR_GFP–RFP_ and the SYNV N, P, and L protein have been described previously (Ganesan et al., 2013). Agrobacterial cell cultures were resuspended in buffer containing 10 mM MgCl_2_, 100 μM acetosyringone, and 10 mM MES, pH 5.6, followed by incubation for 2–4 h at room temperature. Before infiltration, appropriate volumes of different agrobacterial strains were mixed to reach a final OD_600_ of 0.2 for each strain unless otherwise stated. The bacterial cell mixtures were infiltrated into leaves of *N. benthamiana* plants at the five–six-leaf stage with a needleless 1-ml syringe. The infiltrated leaves were monitored with a Zeiss SteREO Lumar V12 stereo fluorescence microscope with a Lumar 31 filter set for RFP detection (excitation, 565/30 nm; emission, 620/60 nm) and a Lumar 38 filter set for GFP detection (excitation, 470/40; emission, 525/50).

### Protein Analysis

Total protein samples were extracted from infiltrated leaf tissues and separated by 12.5 or 6% (for L protein detection only) sodium dodecyl sulfate–polyacrylamide gel electrophoresis (SDS-PAGE). The gels were transferred to polyvinylidene difluoride membranes, and the blots were detected with monoclonal antibodies against GFP (Abcam), Actin (Sangon Biotech), or polyclonal antibodies specific to RFP (Abcam). RSV N- or L-specific antisera were generated in our laboratory, and the detailed information will be described elsewhere. The protein blots were also stained with Ponceau S to visualize the Rubisco large subunit.

## Results

### Construction of RSV vRNA1-Based MR

**FIGURE 1:**
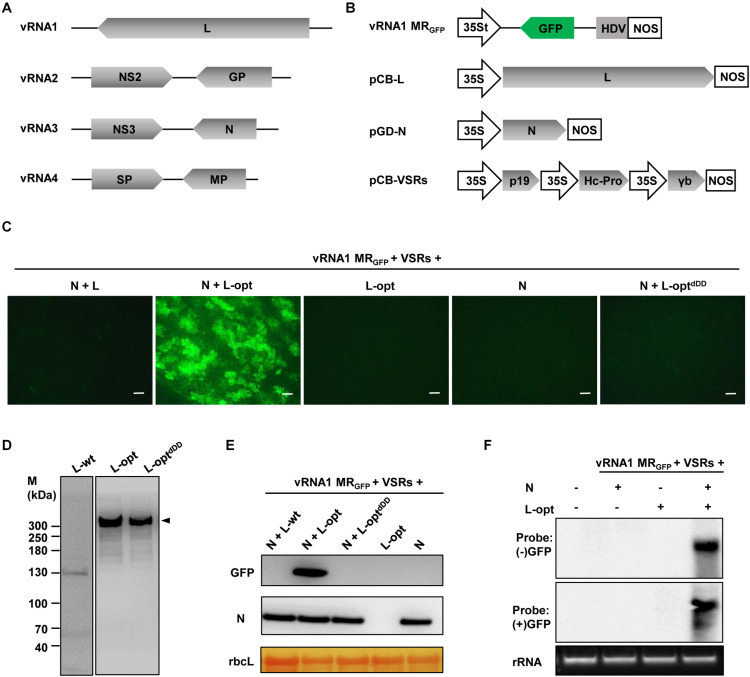
Establishment of a rice stripe virus (RSV) RNA1-based minireplicon (MR). **(A)** Schematic representation of the RSV genome structure. **(B)** Illustration of the binary plasmid engineered to transcribe a vRNA1-based MR_GFP_, in which the virion-sense L coding region was replaced with a green fluorescent protein (GFP) gene. Additional illustrations show the supporting binary plasmids designed to express the N protein and the wild-type or codon-optimized (L-opt) L proteins, and the pCB-VSRs plasmid for simultaneous expression of the tomato bushy stunt virus p19, barley mosaic stripe virus γb, and tobacco etch virus Hc-Pro viral suppressors of RNA silencing (VSRs). 35S, 35S promoter; 35St, a truncated version of the 35S promoter (Wang et al., 2015); HDV, hepatitis delta virus ribozyme; NOS, nopaline synthase terminator. **(C)** GFP foci in *Nicotiana benthamiana* leaves coexpressing vRNA1 MR_GFP_, VSRs, and N and/or L proteins as indicated above each panel. Agroinfiltrated leaves were photographed with a fluorescence microscope at 5 days post-infiltration (dpi). Scale bar = 200 μm. **(D)** Detection of L protein derivatives with L-specific antiserum following transient expression via agroinfiltration. Protein size markers (M) are labeled on the left, and the arrowhead on the right indicates the product corresponding to the full-length L protein (∼337 kDa). **(E)** Western blot analyses of the GFP and N proteins expression in infiltrated leaf tissues. The large RuBisCO (rbcL) subunit was stained by Ponceau S to indicate equal protein loading. **(F)** Northern blot analyses of the replication and transcription products derived from vRNA1 MR_GFP_. The blots were hybridized with both sense (+) and antisense (−) GFP probes.

As a first step toward developing the RSV reverse genetic system, we began with construction of the negative-sense RNA1-based MR. For this purpose, we cloned the vRNA1 cDNA and replaced the L ORF with a GFP ORF. This MR cDNA was inserted into a binary plasmid between a truncated version of the cauliflower mosaic virus 35S promoter (35St) and a hepatitis delta virus (HDV) ribozyme sequence to produce the vRNA1 MR_GFP_ transcription plasmid. The 35St promoter has a truncation of the sequence downstream of the transcription initiation site (Wang et al., 2015) and generates virion-sense MR transcripts with authentic 5′ terminus, and precise 3′ viral RNA end was obtained with HDV ribozyme cleavage (Figure 1B). For members in the order *Bunyavirales*, the N and L proteins constitute the minimal viral factors required for genome encapsidation, replication, and transcription (Bouloy and Flick, 2009). Therefore, mixtures of agrobacterial cultures harboring the vRNA1 MR_GFP_ plasmid and the binary plasmids designed for expression of RSV N and L proteins were used to infiltrate *N. benthamiana* leaves. Three viral suppressors of RNA silencing (VSRs), i.e., tomato bushy stunt virus p19, barley mosaic stripe virus γb, and tobacco etch virus Hc-Pro, were also included in the infection mixtures because these VSRs have been proven to be beneficial for rescue of other plant NSV MRs (Ganesan et al., 2013; Wang et al., 2015; Gao et al., 2019; Feng et al., 2020a; Verchot et al., 2020). Despite repeated attempts and modifications of the protocol, no GFP expression was observed throughout the infiltrated leaf patches (Figure 1C, leftmost panel), indicating that active RNPs were not reconstituted *in vivo*.

Since RSV is a cytoplasmic virus that does not have a nuclear phase during the natural infection cycle, we suspected that plasmid-based nuclear transcription of the unusually long L messenger RNA (mRNA) (∼8.8 kb) by RNA polymerase II might incur aberrant mRNA splicing that could affect the integrity and level of L protein expression. Indeed, following transient expression after agroinfiltration, immunoblot analysis using the L protein-specific antiserum failed to detect the full-length L protein with a predicted molecular weight of 337 kDa. Instead, several aberrant faster-migrating products were observed, with a predominant product of about 130 kDa (Figure 1D, left panel). To alleviate this problem, we optimized the L coding sequence using algorithms designed to maximize *N. benthamiana* codon usage and to remove cryptic intron splicing sites and potential premature transcription termination signals. Upon transient expression, the optimized L gene (L-opt) resulted in abundant expression of the full-length L protein with minimal truncation products (Figure 1D, middle panel). More importantly, in *N. benthamiana* leaves infiltrated with mixtures of *Agrobacterium* cultures containing constructs for the L-opt, N, vRNA1 MR_GFP_, and the three VSRs, discrete GFP foci were clearly visible starting from 4 days after infiltration (dpi), and GFP expression persisted for more than 2 weeks (Figure 1C). Because the negative-sense vRNA1 MR_GFP_ transcripts should not permit direct translation of the GFP ORF encoded on the complementary strand, the presence of GFP signals suggests functions mediated by the coexpressed N and L-opt proteins. This assessment was corroborated by control experiments in which no GFP expression was detected when either the N or L-opt supporting binary plasmids were omitted. An additional control experiment with a catalytically inactive L mutant (L-opt^dDD^) also confirmed the requirement of the L enzymatic activities for MR activity (Figure 1C). This L-opt^dDD^ mutant carried a deletion of the double aspartic acid residues of the conserved SDD motif that is essential for bunyavirus polymerase activities (Jin and Elliott, 1992; Albarino et al., 2009). MR reporter gene expression visualized by fluorescence imaging was also verified by immunoblotting (Figure 1E). To further confirm vRNA1 MR_GFP_ replication and transcription, Northern hybridizations were performed to analyze RSV RNA species in agroinfiltrated tissues at 6 dpi. As shown in Figure 1F, GFP-specific probes detected the presence of both the positive- and negative-sense viral RNAs in total RNA samples only when leaf tissues were infiltrated to express the N, L-opt, VSRs, and vRNA1 MR_GFP_. We note that the (−) strand GFP probe was designed to detect the positive-sense replication products (cRNA) and the transcription products (GFP mRNA) of vRNA1 MR_GFP_, which are about the same length (Wu et al., 2013).

In attempts to optimize the RSV MR system by altering the relative ratios of the supporting N and L proteins, the concentration of *Agrobacterium* cultures harboring the N- or the L-opt constructs were increased gradually from OD_600_ 0.2 to 1.0, while the remaining bacterial strains in the *Agrobacterium* mixtures were maintained at OD_600_ 0.2. The highest MR activities were observed when both the N and L bacterial strains were present in the mixtures at 0.2 OD_600_ concentrations. Increasing the expression of either protein decreased GFP expression progressively (Supplementary Figure 2). In summary, the above results convincingly show that the L gene codon optimization led to improved expression of functional L protein to levels sufficient to support efficient vRNA1-based MR replication and transcription.

### Development of vRNA2 and vRNA3-Based Ambisense MRs

**FIGURE 2:**
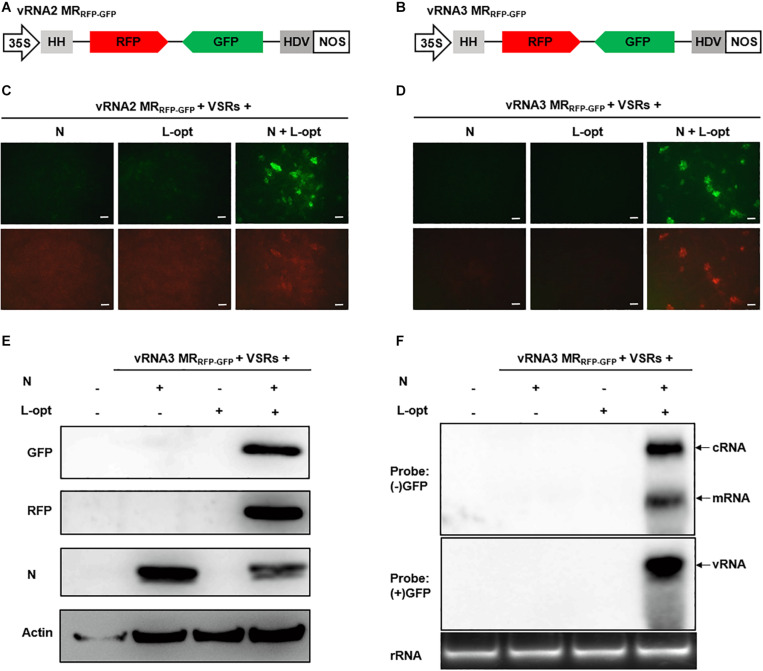
Development of rice stripe virus (RSV) vRNA2- and vRNA3-based MR systems. **(A, B)** Schematic representation of the constructs used for transcription of **(A)** vRNA2 MR_RFP__–__GFP_ and **(B)** vRNA3 MR_RFP__–__GFP_. The virion- and complementary-strand viral open reading frames (ORFs) in RNA2 and RNA3 were replaced with red fluorescent protein (RFP) and green fluorescent protein (GFP), respectively. **(C, D)** Visualization of GFP and RFP expression from **(C)** vRNA2 MR_RFP__–__GFP_ and **(D)** vRNA3 MR_RFP__–__GFP_ supported by N, L-opt, or N plus L-opt. Agroinfiltrated leaves were photographed with a fluorescence microscope at 8 dpi. Scale bar = 200 μm. **(E)** Western blot analyses of the expression of the GFP, RFP, and N proteins in agroinfiltrated leaf tissue shown in **(D)**. The blot was probed with a monoclonal antibody against *N. benthamiana* Actin protein to serve as a protein loading control. **(F)** Northern blot detection of the virion-sense (v)RNA, complementary-sense (c)RNA, and the subgenomic messenger (m)RNA derived from vRNA3 MR_RFP__–__GFP_ replication and transcription with sense (+) and antisense (−) GFP probes.

Unlike the negative-sense RNA1, RSV RNA2–4 employ an ambisense coding strategy in which the vRNA and the cRNA each encode an ORF (Figure 1A). To engineer ambisense MRs, we cloned the RNA2 and RNA3 cDNAs and substituted GFP and RFP ORFs for the complementary-sense and the virion-sense viral ORFs, respectively. The cDNAs of the two virion-sense MRs were fused to a 5′ hammerhead (HH) ribozyme and 3′ HDV ribozyme for terminal sequence processing, and the cassettes were positioned downstream of the 35S promoter in a binary vector to generate vRNA2 MR_RFP__–__GFP_ and vRNA3 MR_RFP__–__GFP_ (Figures 2A,B). MR reporter expression assays were conducted similarly to those of the vRNA1 MR_GFP_ assays described above. GFP cell foci were observed only when leaves were agroinfiltrated to express the N and L-opt proteins together with vRNA2 MR_RFP__–__GFP_ or vRNA3 MR_RFP__–__GFP_, and these GFP-positive cells also supported relatively weak RFP expression (Figures 2C,D). Notably, in the case of vRNA2 MR_RFP__–__GFP_, we observed minor amounts of RFP but not GFP background expression in the absence of the N or L proteins (Figure 2C, lower panels). This leaky RFP expression suggests that the primary vRNA2 MR_RFP__–__GFP_ transcripts permit limited ribosomal translation of the 5′-proximal RFP ORF despite the absence of a 5′ cap structure in the ribozyme processed transcripts. The vRNA3-based MR was subjected to further molecular analyses in which immunoblot assays confirmed MR-derived GFP and RFP expression only in the presence of the coexpressed N and L-opt proteins (Figure 2E). In Northern blot assays, the (−) strand GFP probe hybridized with cRNAs representing the full-length replication products and the smaller subgenomic GFP mRNA resulting from viral transcription. When the (+) strand GFP probe was used for hybridization, a specific band corresponding to vRNA3 MR_RFP__–__GFP_ was evident in RNAs extracted from leaf tissues expressing the N and L-opt proteins (Figure 2F). Altogether, these results support a model whereby primary virion-sense MR transcripts were encapsidated and replicated by the N and L proteins, and both the vRNAs and cRNAs were templates for transcription of subgenomic mRNA species.

### Effects of *cis*-Element Deletions on Minireplicon Reporter Gene Expression

**FIGURE 3:**
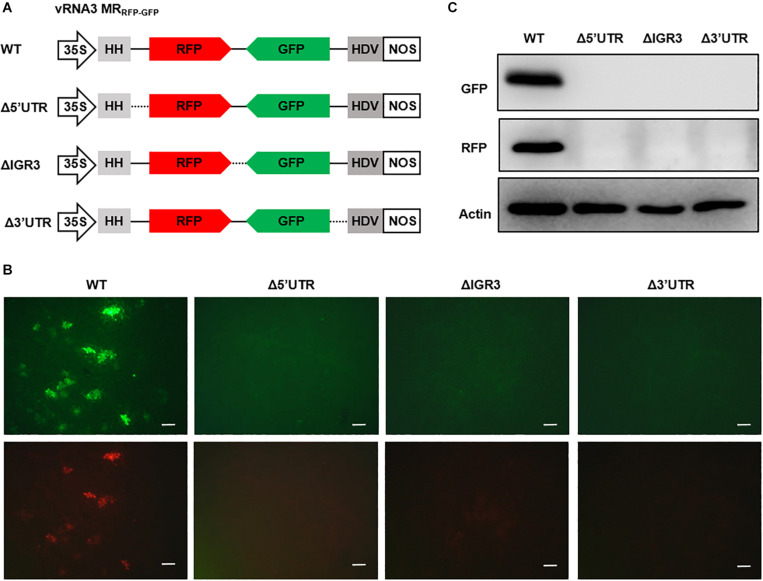
Reverse genetics analysis of the roles of *cis*-elements in vRNA3 MR_RFP__–__GFP_ reporter gene expression. **(A)** Schematic representations of vRNA3 MR_RFP__–__GFP_ and its mutant derivatives. Δ5′ untranslated region (UTR), Δ3′ UTR, and ΔIGR3 indicated the 5′ or 3′ untranslated regions, or the intergenic region were deleted in vRNA3 MR_RFP__–__GFP_, respectively. Solid lines denote viral non-coding regions, and dashed lines indicate the sequence regions being deleted. **(B)** GFP foci in *N. benthamiana* leaves agroinfiltrated to express the N, L-opt, VSR, and vRNA3 MR_RFP__–__GFP_ or its mutant derivatives. Images were taken at 8 dpi with a fluorescence microscope. Scale bar = 200 μm. **(C)** Protein gel blot analysis of GFP and RFP expression in agroinfiltrated leaf tissues. Blot was also probed with Actin-specific antibody to serve as a loading control.

The 5′ and 3′ UTRs of bunyavirus genomic segments contain conserved terminal complementary sequences that can form characteristic “panhandle” structures that serve as signals for genome encapsidation and as promoters for viral replication and transcription (Barr et al., 2003). For ambisense genomic segments, additional *cis*-acting elements located in the IGR function to terminate transcription of mRNAs encoded by the two convergent ORFs (Ikegami et al., 2007; Guu et al., 2012; Wu et al., 2013). After engineering the RSV ambisense MRs, we set out to test the role of *cis*-elements in MR reporter gene expression by using the vRNA3 MR_RFP__–__GFP_ as an example. We generated three MR mutants, each with a deletion in the 5′ UTR, 3′ UTR, or IGR sequences (Figure 3A). When coexpressed with the N and L-opt proteins in *N. benthamiana* leaves, the wild-type vRNA3 MR_RFP__–__GFP_ supported GFP and RFP expression, but the three MR mutants failed to do so (Figures 3B,C). The data based on these reverse genetics analyses verify that the 5′ UTR, 3′ UTR, and IGR non-coding regions are required for MR activity.

### Specific Inhibitory Effect of NS3 on RSV MR Reporter Gene Expression

**FIGURE 4:**
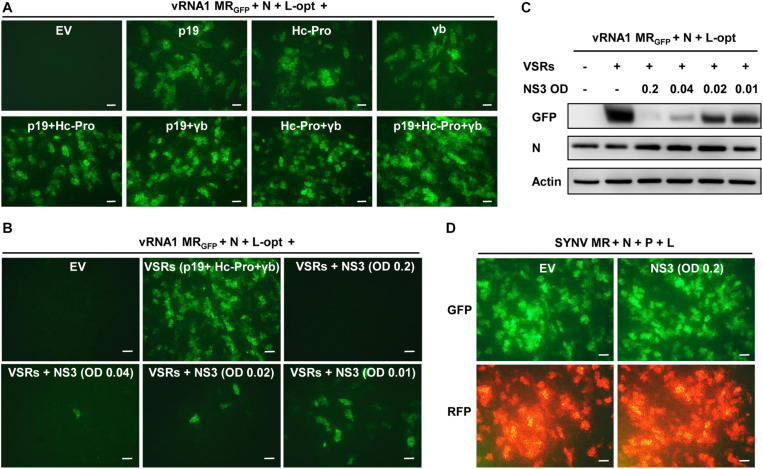
NS3 inhibition of rice stripe virus (RSV) minireplicon (MR) activity. **(A)** Effects of the coexpressed individual and combinatorial heterologous viral suppressor of RNA silencing (VSRs) on RSV MR reporter gene expression. Appropriate volumes of different agrobacterial strains harboring the vRNA1 MR_GFP_ plasmid, the N and L-opt expression plasmids, and either an empty binary vector (EV) or plasmids for expression of a single or multiple VSRs (p19, Hc-Pro, and γb) were mixed to reach a final OD_600_ of 0.2 for each strain. *N. benthamiana* leaves were infiltrated with the bacterial mixtures, and expression of green fluorescent protein (GFP) was photographed at 8 dpi with a fluorescence microscope. **(B,C)** Dose-dependent inhibition of RSV MR activity by NS3 protein. Agrobacterial strains harboring the NS3 expression plasmid or an empty vector (EV) was added into the above MR mixtures containing all three VSRs (p19 + Hc-Pro + γb) at a final OD_600_ of 0.2, 0.04, 0.02, or 0.01. **(B)** GFP foci in agroinfiltrated *N. benthamiana* leaves were imaged at 8 dpi and **(C)** expression of GFP and N proteins in these leaf tissues were detected by Western blotting with anti-GFP and anti-N antibodies. Detection with an anti-Actin antibody was used as a protein loading control. **(D)** Lack of inhibitory effect of NS3 on Sonchus yellow net virus (SYNV) MR activity. SYNV MR assays were carried out by agroinfiltration of *N. benthamiana* leaves to express SYNV MR_GFP–RFP_, the SYNV N, P, and L proteins, VSRs, and an EV control or RSV NS3. The final concentrations of each bacterial strain were adjusted to OD_600_ 0.2. Expression of reporter genes was monitored at 7 dpi under the GFP channel (upper) and RFP channel (bottom). Scale bar = 200 μm in **(A,B,D)**.

The MR assays described above involved coexpression of three heterologous VSRs (p19, γb, and Hc-Pro). We tested whether these VSRs are required for MR activity using the vRNA1 MR_GFP_ as a model. As shown in Figure 4A, when all three VSRs were omitted in the *Agrobacterium* mixtures, GFP expression from vRNA1 MR_GFP_ was not detected. In contrast, expression of each of the three VSRs supported MR reporter gene expression. Combinatorial expression of two VSRs further enhanced GFP expression, and the highest MR activity was observed when all three VSRs were coexpressed. These experiments indicated that RSV MR reporter gene expression critically relies on suppression of host RNA silencing and that individual suppressors have additive effects on MR activity. RSV-encoded NS3 also acts as a potent VSR through sequestration of double-stranded RNA (Xiong et al., 2009; Shen et al., 2010). We next tested whether NS3 might have a similar VSR function. Interestingly, coexpression of NS3 together with the three heterologous VSRs led to marked suppression of vRNA1 MR_GFP_-derived GFP expression, and the inhibitory effects of NS3 appeared to be dose dependent. Even when *Agrobacterium* cultures carrying the NS3 plasmid were diluted by 20-fold to a final OD_600_ of 0.01, the inhibition was still apparent (Figures 4B,C). In contrast, NS3 had no discernible effect on reporter gene expression of an SYNV-based MR supported by the SYNV N, P, and L proteins (Figure 4D), suggesting that the NS3 repressive role was specific to RSV-based MR.

### Development of Intercellular Movement-Competent MR Systems

**FIGURE 5:**
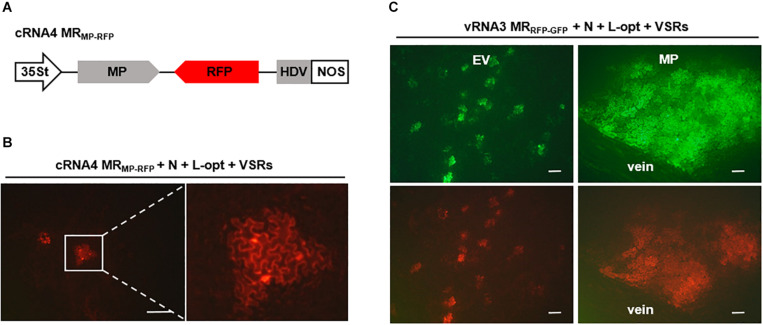
Cell-to-cell movement of *in vivo* reconstructed MR ribonucleoprotein (RNP) assisted by the rice stripe virus (RSV) movement protein (MP). **(A)** Schematic representation of RSV cRNA4-based MR_MP–GFP_. The SP coding region in the complementary-strand of RNA4 was replaced by red fluorescent protein (RFP). **(B)** Images showing cell clusters with RFP signals in *N. benthamiana* leaves that were agroinfiltrated to express the N and L proteins, viral suppressors of RNA (VSRs), and cRNA4 MR_MP–RFP_. The boxed sector is magnified to highlight the movement of RNA4 RNP. **(C)** Visualization of fluorescent reporter proteins expressed from the reconstructed vRNA3 MR_RFP__–__GFP_ in the absence or presence of the RSV MP. Leaves of *N. benthamiana* plants were infiltrated with mixtures of agrobacterial cultures harboring the vRNA3 MR_R__FP__–__GFP_ plasmid, the N- and L-opt expression plasmids, and either an empty binary vector (EV) or an RSV MP expression vector. Infiltrated leaves were photographed at 10 dpi with a fluorescence microscope. Scale bar = 200 μm.

It has been shown that cell-to-cell movement of negative-stranded RNA plant rhabdoviruses involves the passage of RNPs through plasmodesmata that is facilitated by viral MPs (Wang et al., 2015; Zhou et al., 2019). To determine whether *in vivo* reconstituted RSV RNPs capable of replication and transcription can move from cell to cell, we constructed a cRNA4-based MR, termed cRNA4 MR_MP–RFP_, in which the MP ORF was retained, whereas the SP ORF was replaced by an RFP gene (Figure 5A). Upon coexpression with the N, L-opt, and VSRs, active RNPs were reconstituted as evidenced by RFP expression. However, unlike the single-cell fluorescent foci observed in the RNA1–3-based MRs described above (Figures 1, 2), the cRNA4 MR_MP–RFP_ leaf tissues developed multicell clusters indicative of RNP cell-to-cell movement (Figure 5B). To further test whether the RSV MP could facilitate intercellular trafficking of RNPs *in trans*, we reconstituted RNPs containing vRNA3 MR_RFP__–__GFP_ in the absence or presence of the RSV MP. As anticipated, fluorescent reporter proteins expressed from reconstituted RNPs lacking the RSV MP were confined to discrete single cell foci. In contrast, when the RSV MP was expressed ectopically, fluorescence spread throughout the mesophyll tissues but failed to enter the leaf veins (Figure 5C). Thus, these data show that RSV MP promotes RNP cell-to-cell movement both *in cis* and *in trans* but not vascular movement, although we cannot exclude the remote possibilities that RSV MP facilitated RNP reconstitution or viral RNAs movement to neighboring cells.

### Development of cRNA-Based MR Systems That Bypass the Necessity for Supporting N and L Plasmids

**FIGURE 6:**
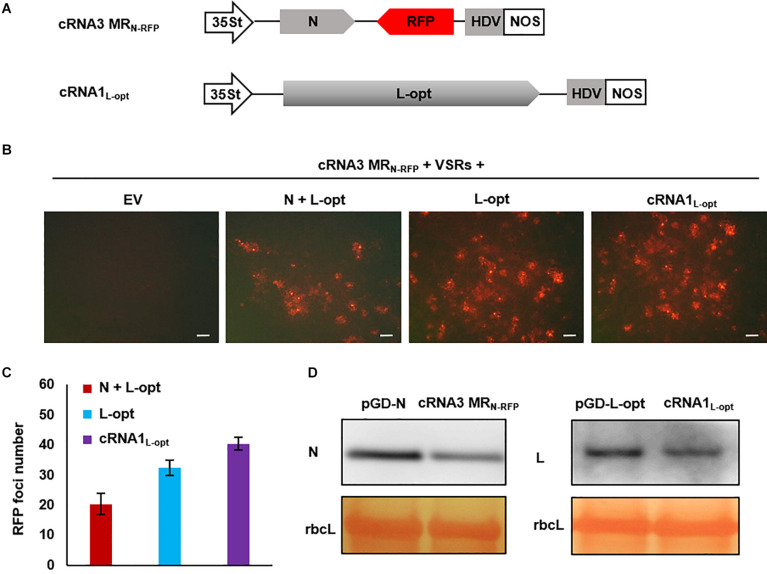
Development of an autonomous minireplicon (MR) system without the need of the N and L expression plasmids. **(A)** Schematic representations of the binary constructs designed for transcription of cRNA3 MR_N–RFP_ and cRNA1_L–opt_. The cRNA3 MR_N–RFP_ construct contains a red fluorescent protein (RFP) gene substituted for the NS3 coding region in the complementary-strand of RNA3, and the cRNA1_L–opt_ plasmid encodes the codon-optimized L protein sequence (L-opt). **(B)** Visualization of RFP expression in *N. benthamiana* leaves agroinfiltrated to express the N and L-opt proteins, the L-opt protein alone, cRNA1_L–opt_, or an empty binary plasmid (EV). Infiltrated leaves were photographed at 8 dpi with a fluorescence microscope. Scale bar = 200 μm. **(C)** Quantitative analyses of the number of RFP foci per field in infiltrated leaf tissues. The data show the means of six independent micrographs (*n* = 6), and the error bars denote standard deviation (±SD). **(D)** Comparison of the expression levels of N and L in infiltrated leaves. Total protein was extracted from agroinfiltrated leaves with the indicated constructs in the presence of viral suppressor of RNAs (VSRs) at 2 dpi and detected with N- and L-specific antiserums. The large RuBisCO (rbcL) subunit was stained by Ponceau S to visualize protein loading.

For segmented bunyaviruses, both the N and L nucleocapsid proteins are encoded by the 5′-proximal ORFs in the cRNAs. Thus, plasmid-derived cRNA transcripts might be used directly as templates to translate the N and L proteins. Based on this assumption, we generated a cRNA3 MR_N–RFP_ transcription plasmid in which the N ORF in cRNA3 was retained and the NS3 was replaced by the RFP ORF. In addition, we also generated a cRNA1 binary construct termed cRNA1_L–opt_ where the optimized L coding sequence was substituted for the original sequence (Figure 6A). When assayed for MR reporter expression, cRNA3 MR_N–RFP_ alone failed to express the RFP protein; however, cells with red fluorescence were readily detected when the N and L proteins or only the L protein were supplied from the codelivered binary plasmids (Figure 6B). This result suggests that the N protein was generated from cRNA3 MR_N–RFP_ in sufficient amounts to support MR replication. In fact, quantitative analysis of the number of RFP foci revealed that the additional N protein provided *in trans* from the N supporting plasmid appeared to have a deleterious effect on cRNA3 MR_N–RFP_ activity (Figure 6C). Similarly, we found that the L protein provided from cRNA1_L–opt_ supported cRNA3 MRN-RFP replication and transcription (Figure 6B). In this case, the infiltrated leaves contained a greater number of RFP foci than those appearing when the L protein was supplied *in trans* from the expression plasmid (Figure 6C). Immunoblot analyses verified that cRNA3 MR_N–RFP_ and cRNA1_L–opt_ permitted translation of the N and L proteins, respectively, although the protein levels were slightly lower than those expressed from the binary expression plasmids (Figure 6D). Therefore, the cRNA1- and cRNA3-based MR systems have the merits of higher activity and fewer plasmids required for delivery.

### Generation of a Multicomponent System Capable of Autonomous Replication and Cell-to-Cell Movement

**FIGURE 7:**
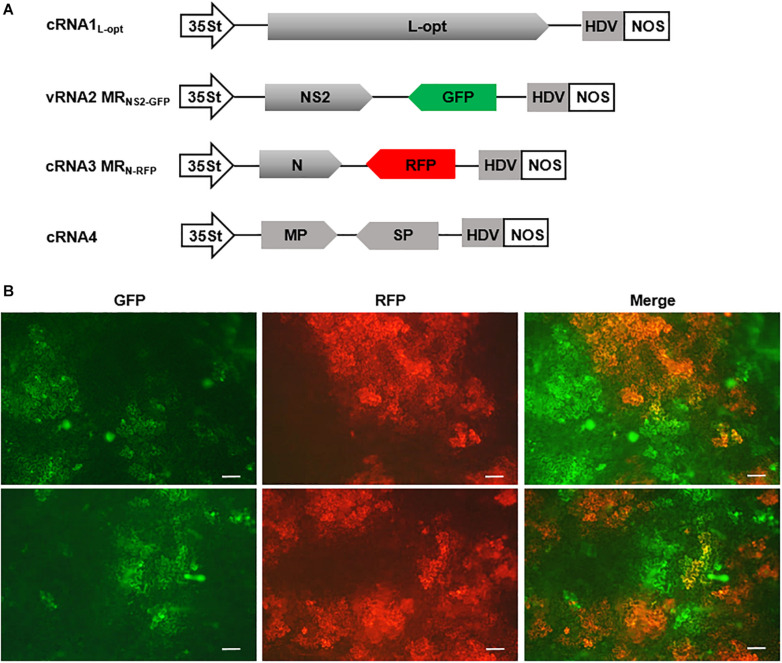
A four-component system capable of autonomous replication and intercellular movement. **(A)** Diagrams showing the constructs designed for transcription of cRNA1_L–opt_, vRNA2 MR_NS__2__–GFP_, cRNA3 MG_N–RFP_, and cRNA4. vRNA2 MR_NS__2__–GFP_ contains a green fluorescent protein (GFP) reporter gene substitution for the GP gene, whereas cRNA3 MG_N–RFP_ carries a red fluorescent protein (RFP) gene in place of the NS3 gene. **(B)** Visualization of GFP and RFP expression in *N. benthamiana* leaves infiltrated with mixtures of agrobacterial suspensions harboring the binary constructs shown in **(A)** and the viral suppressor of RNAs (VSRs) plasmid. The top and bottom panels show fluorescence in two representative leaves. Scale bar = 200 μm.

Having established MR systems based on the four RSV segments, we next attempted to generate a multicomponent system containing all four genome segments. In addition to the cRNA3 MR_N–RFP_ and cRNA1_L–opt_ constructs described above, we also generated a cRNA4 plasmid and a vRNA2 MR_NS__2__–GFP_ construct in which the GP gene was replaced by GFP. Within these constructs, viral cDNAs were inserted between the 35St promoter and HDV ribozyme sequence to generate capped transcripts with precise termini (Figure 7A). We delivered this minimal set of four plasmids along with the plasmid encoding the three VSRs into *N. benthamiana* leaves via agroinfiltration. The N and L expression plasmids were not included because the two proteins can be translated from the cRNA3 MR_N–RFP_ and cRNA1_L–opt_ transcripts, as shown in Figure 6. The GP gene in RNA2 and the NS3 gene in RNA3 were replaced by GFP and RFP to facilitate tracking of rescue events in plants. This design was based on the following assumptions: (i) The GP gene is not essential for plant infections because enveloped RSV particles have never been observed (Falk and Tsai, 1998), and a recombinant TSWV mutant lacking the GP gene has been recovered and displayed no defects in plant infections (Feng et al., 2020a); (ii) NS3 may be dispensable for RSV infections because the analogous NSs genes from many mammalian bunyaviruses and TSWV can be deleted or substituted by reporter genes without affecting virus replication (Bridgen et al., 2001; Blakqori and Weber, 2005; Ikegami et al., 2006; Ogawa et al., 2007; Elliott et al., 2013; Rezelj et al., 2015; Brennan et al., 2017; Alexander et al., 2020; Feng et al., 2020a; Oymans et al., 2020; Woelfl et al., 2020). At 8 dpi, GFP and RFP fluorescence appeared in infiltrated leaf tissues and spread extensively to form large cell clusters, which suggests that the four-component system is capable of autonomous replication and cell-to-cell movement. However, cells with green and red fluorescence appeared to display significant mutual exclusion because only a small proportion of the fluorescing cells expressed both reporter proteins (Figure 7B). These fluorescent signals never spread into vascular tissues or the upper non-inoculated leaves, and reverse transcription PCR and immunoblotting also failed to detect systemic RSV infections (data not shown). Multiple attempts involving alteration of the ratios of the components, reintroduction of the GP and/or NS3 genes into the vRNA2 MR_NS__2__–GFP_ and cRNA3 MR_N–RFP_ constructs to convert them to wild-type RNA2 and RNA3, or supply of additional N and L proteins *in trans* from expression plasmids, etc., have also been unsuccessful in recovery of recombinant RSV from non-inoculated leaves (data not shown).

## Discussion

To establish a reverse genetic system for an NSV, it is necessary to reconstitute active RNPs *in vivo* by expression of nucleocapsid core proteins and viral RNAs. In this study, we exploited the fact that RSV can systemically infect *N. benthamiana*, a model plant highly amenable to agroinfiltration-mediated transient expression. To test rescue of RSV MRs, we designed *Agrobacterium* binary plasmids for expression of the RSV N and L proteins and an RNA1-based MR. After agroinfiltration of *N. benthamiana* leaves to deliver these constructs, the small N protein accumulated to a high level, but the full-length large L protein was not detected. Consequently, MR activity was not detected. It is known that pre-mRNAs synthesized by RNA polymerase II (Pol II) are cotranscriptionally spliced through coordination of the carboxy-terminal domain of Pol II (Fong and Bentley, 2001; Kornblihtt et al., 2004). This can present an issue for many cytoplasmic replicating NSVs whose RNAs may contain potential splicing signals. A solution to this problem was first described during the rescue of Crimean Congo hemorrhagic fever virus (CCHFV), a bunyavirus in the family *Nairoviridae*. Transcription of the CCHFV L protein mRNA by a Pol II promoter resulted in truncated protein products that failed to initiate recombinant infection, but elimination of cryptic splicing sites from the L mRNA through codon optimization resulted in functional L protein translation and successful virus recovery (Bergeron et al., 2015). A similar strategy was used to generate recombinant TSWV from plasmids, whose L and GP genes were predicted to contain numerous intron-splicing sites that interfered with 35S promoter-driven protein expression and virus rescue (Feng et al., 2020a). Based on these studies, we optimized the RSV L codon usage to remove potential splicing sites and premature termination signals. This strategy markedly improved the expression of the ∼337 kDa full-length L protein and resulted in efficient vRNA1-based MR reporter gene expression. Notably, the sizes of the L gene of CCHFV (11.8 kb), RSV (8.8 kb), and TSWV (8.6 kb) are the largest among the NSVs^1^. Together, these studies highlight problems that may be encountered during expression of large viral proteins of cytoplasmic NSVs and underscore the magnitude of the splicing issue.

We next constructed vRNA2- and vRNA3-based ambisense MRs that encode two reporter genes in opposite orientations. Both reporters were expressed from the MRs only when supported by the coexpressed N and L proteins, and MR activities were confirmed by detection of genomic RNAs and subgenomic mRNAs. During bunyavirus RNA synthesis, the complementary terminal UTR sequences form a panhandle-like structure that functions as a promoter for L-protein-mediated replication and transcription (Barr and Wertz, 2004; Guu et al., 2012). Termination of ambisense mRNA transcription is controlled by specific sequence motifs and secondary structures in the IGRs (Ikegami et al., 2007). Mapping of the 3′ termini of RSV ambisense mRNAs revealed a AUCCGGAU nucleotide sequence within the IGR hairpin secondary structures that potentially functions as a termination signal during mRNA transcription (Wu et al., 2013). To determine the importance of the sequence, we conducted deletion analyses with the RSV RNA3 MR that confirmed the roles of the IGR in MR mRNA transcription. Together, these data suggest that the MR systems developed in this study are suitable for analyses of the roles of *cis*-acting viral elements in viral replication and transcription.

Use of the MR systems revealed a specific inhibitory role of the NS3 VSR in RSV MR reporter gene expression, although minimizing RNA silencing responses by expression of the p19, Hc-Pro, and γb VSRs was critically important for the MR activity. NS3 inhibition was found to be highly active because titration of the *Agrobacterium* strain to OD_600_ 0.01 still resulted in substantial reduction in MR activity. Inhibition of MR reporter gene expression has also been documented for the analogous NSs virulence proteins encoded by Bunyamwera and La Crosse orthobunyaviruses (Weber et al., 2001; Blakqori et al., 2003) and thrombocytopenia syndrome Phlebovirus (Brennan et al., 2015). In contrast, The Rift Valley fever Phlebovirus NSs has been reported to enhance MR activity (Ikegami et al., 2005). The situation with TSWV NSs appeared to be more complicated because TSWV NSs expression boosted the activity of the small segment-derived MR, but ectopic expression of NSs abolished the rescue of infectious virus (Feng et al., 2020a). In the case of Bunyamwera virus, increasing the expression of the N, L proteins or MR RNA failed to neutralize the NSs-mediated inhibition, suggesting that the NSs inhibitory role was not due to interference of transient protein/RNA expression (Weber et al., 2001). We have shown that transient expression of the RSV N protein was not affected by the coexpressed NS3. Additionally, RSV NS3 had little effect on SYNV MR activity. These observations argue against any non-specific roles of NS3, such as perturbation of agroinfiltration-mediated transient expression, induction of cell death, or triggering general defense mechanisms. Thus, NS3 could have a specific inhibitory effect on RSV polymerase activity that may warrant further studies.

As the next step toward optimizing the MR systems, we constructed cRNA3 MR_N–RFP_ and cRNA1_L–opt_ to explore autonomous replication without the need to supply the N and L proteins *in trans*. The advantages of this system include higher MR activity and the ability to deliver fewer plasmids. This development, together with the demonstration that RSV MP supported intercellular trafficking of the reconstituted RNPs *in cis* and *in trans*, prompted us to attempt rescues of all the four RSV genome components from plasmids. A minimal set of four constructs were delivered into *N. benthamiana* leaves to express the full-length cRNA1 and cRNA4 and the reporter gene-bearing vRNA2 MR_NS__2__–GFP_ and cRNA3 MR_N–RFP_. In this four-component system, we observed expression and intercellular movement of both fluorescent reporters. Although infectious RSV was not recovered, the patterns of GFP and RFP expression revealed that: (i) RNPs consisting of vRNA2 MR_NS__2__–GFP_ and cRNA3 MR_N–RFP_ were successfully reconstituted as judged from GFP and RFP expression; (ii) cRNA_L–opt_ was expressed in the infiltrated cells and was active in synthesis of L proteins required for reporter gene expression; (iii) cRNA4 was also expressed in the fluorescent cells and functioned to supply the MP protein because the fluorescent signal exhibited extensive cell-to-cell movement; and (iv) in the GFP-expressing cells resulting from successful reconstitution of vRNA2 MR_NS__2__–GFP_ RNP, cRNA3 MR_N–RFP_ must also be active to provide N protein needed for RNP assembly and replication. However, the lack of RFP fluorescence in most of the GFP-expressing cells suggests that active cRNA3 RNP was not reconstituted simultaneously in these cells with functional RNA2 RNP and *vice versa*. A simple conclusion from these analyses is that although all the four RNA derivatives appeared to be expressed in at least some cells within the infiltrated areas, simultaneously reconstitution of all four RNPs in the same cells appears to be a rare event or in insufficient amounts needed for rescue of recombinant virus. The exact reasons for the inability to generate productive RNP assembly will require scrutinization in future studies.

## Conclusion

In summary, we have established RSV MR systems based on the four virion-/complementary-sense genome segments. These MR systems will enable reverse genetics studies of the roles of *cis-*elements and *trans*-acting factors in viral replication, mRNA transcription, and intercellular movement and also will provide a platform for construction of more sophisticated recombinant systems.

## Data Availability Statement

The datasets presented in this study can be found in online repositories. The names of the repository/repositories and accession number(s) can be found below: https://www.ncbi.nlm.nih.gov/genbank/, MW463890; https://www.ncbi.nlm.nih.gov/genbank/, MW463891; https://www.ncbi.nlm.nih.gov/genbank/, MW463892; https://www.ncbi.nlm.nih.gov/genbank/, MW463893.

## Author Contributions

ZL and XZ conceived the project and designed the experiments. XZ, KS, YL, SW, and KW performed the experiments. ZL and XZ analyzed the data and interpreted the results. ZL and XZ wrote the manuscript. All authors contributed to the article and approved the submitted version.

## Conflict of Interest

The authors declare that the research was conducted in the absence of any commercial or financial relationships that could be construed as a potential conflict of interest.

## Footnotes

1 https://viralzone.expasy.org/
