# Supplementary material for: Development of Rice Stripe Tenuivirus Minireplicon Reverse Genetics Systems Suitable for Analyses of Viral Replication and Intercellular Movement
Source: Front Microbiol. 2021 Mar 23;12:655256. doi: 10.3389/fmicb.2021.655256 (PMC8021733; doi:10.3389/fmicb.2021.655256)
Supplement: Supplementary file 1 [file Data_Sheet_1.DOCX]

Supplementary Material

## Supplementary Figures


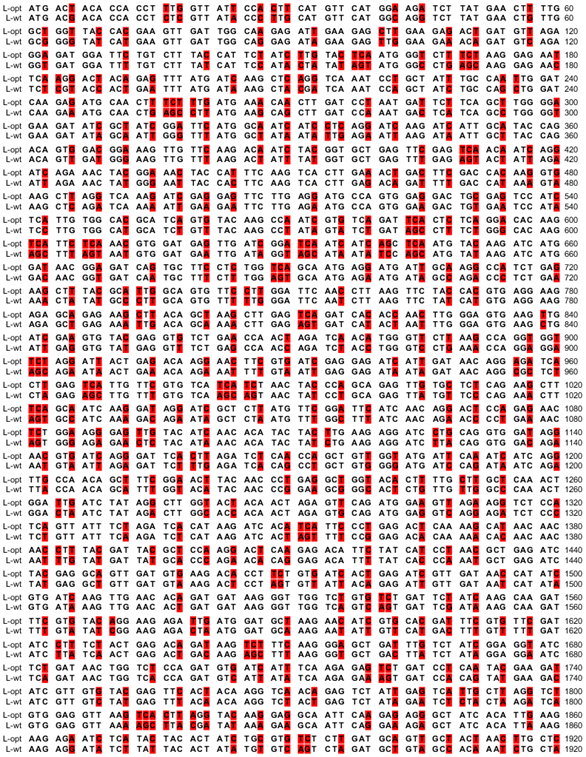
**Supplementary Figure 1.** Alignment of the wild-type (L-wt) and codon-optimized (L-opt) L coding sequences. The nucleotide changes are highlighted in red.


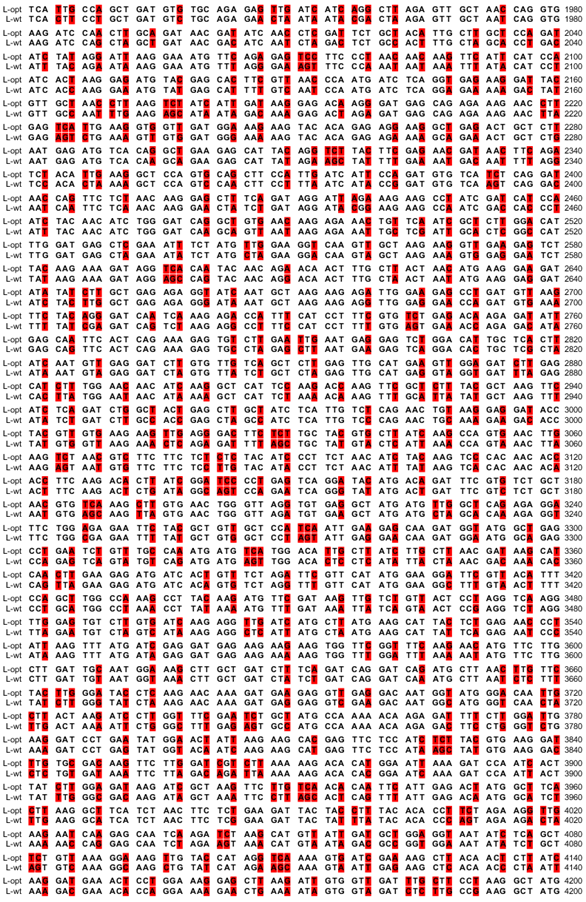


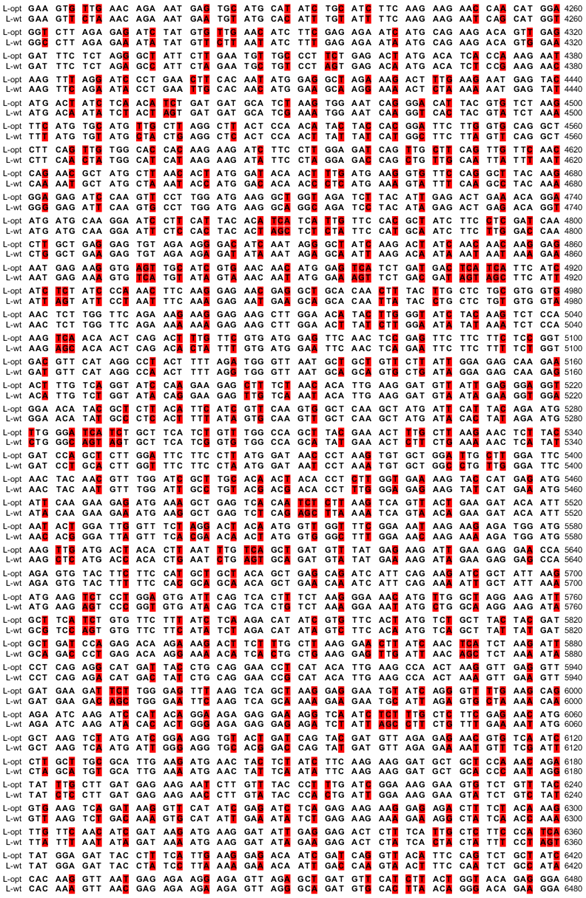


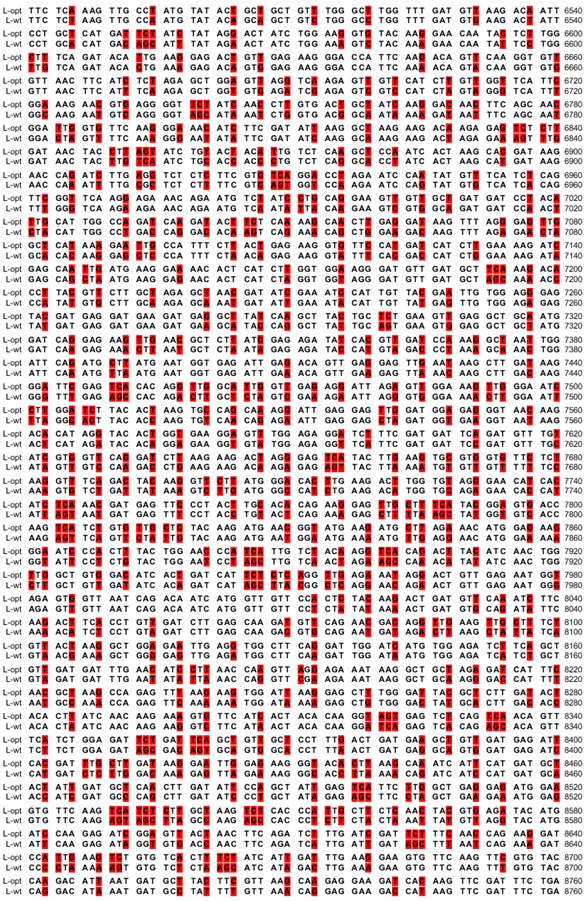


**Supplementary Figure 2.** Determination of the concentration of the N and L proteins needed for optimum vRNA1 MR_GFP_ activity. (A) Visualization of GFP foci in infiltrated leaves. *N. benthamiana* leaves were infiltrated with mixtures of *Agrobacterium* cultures containing the binary vectors for expression of the vRNA1 MR_GFP_, N, L-opt, and VSRs. The final concentrations of vRNA1 MR_GFP_ and VSRs bacterial strains were fixed at OD_600_ = 0.2, whereas the OD_600_ of the N or L strain ranged from 0.2 – 1.0, as indicated above each panel. GFP foci were imaged at 5 dpi with a fluorescence microscope under the GFP channel. Scale bar = 200 μm. (B) Western blot detection of the N and GFP proteins expressed in the leaves shown in (A) using specific antibodies. The Actin blot serves as a protein loading control.


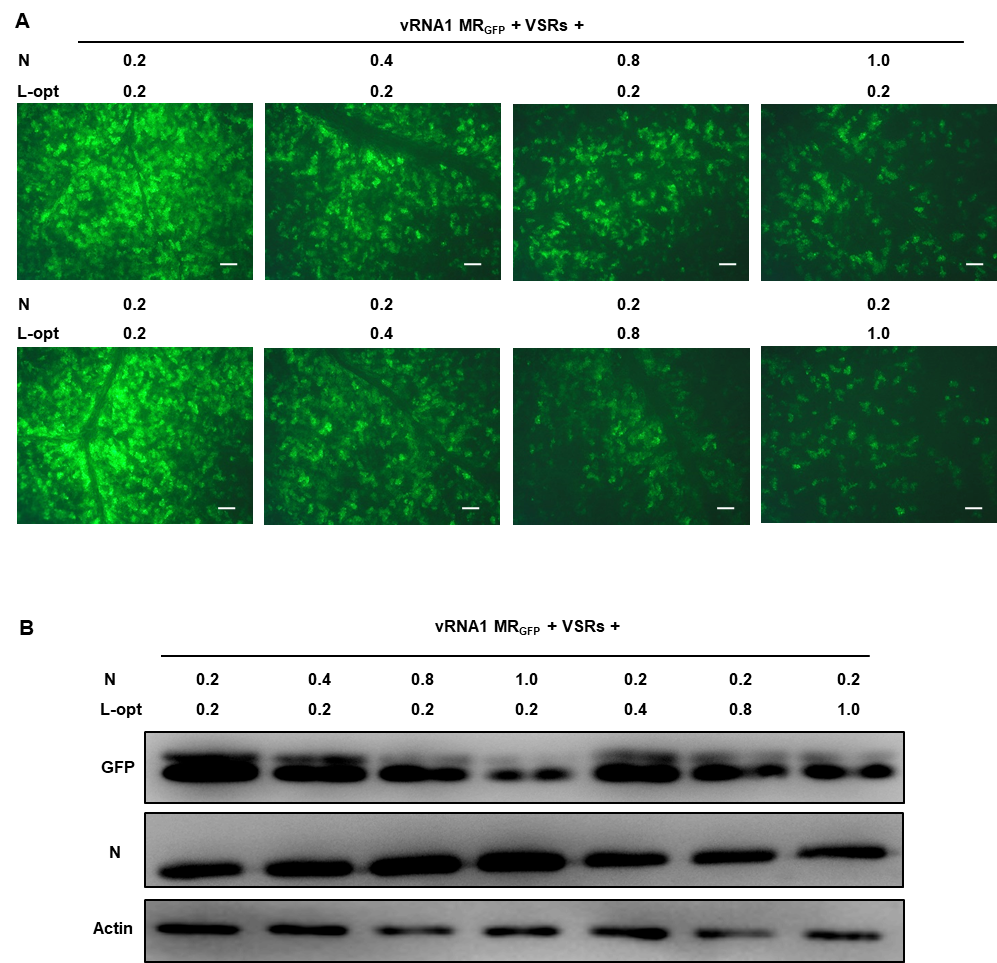


## Supplementary Tables

**Supplementary Table 1.** List of primers used in the study

| Primers | Sequence | Purpose |
| --- | --- | --- |
| L/*Bam*HI/F | GCGGGATCCATGACGACACCACCTCTCGTTATACCCT | To construct pGD-L |
| L/*Sal*I/R | ACGCGTCGACTCAGAAATCGAACTTATGGTCTTCCTCCTGT |  |
| NS3/*Bam*HI/F | ATCTATCTCTGGATCCATGAACGTGTTCACATCGTCTGT | To construct pGD-NS3 |
| NS3/*Sal*I/R | AACGAGCTCTGTCGACCTACAGCACAGCTGGAGAG |  |
| N/*Bam*HI/F | CGCGGATCCATGGGCACCAACAAGCCAGCCA | To construct pGD-N |
| N/*Sal*I/R | ACGCGTCGACCTAGTCATCTGCACCTTCTGCCT |  |
| MP/*Bam*HI/F | ATCTATCTCTGGATCCATGGCTTTGTCTCGACTTTTGTC | To construct pGD-MP |
| MP/*Sal*I/R | AACGAGCTCTGTCGACCTACATGATGACAGAAACTTCAGAT |  |
| pCB/L-opt/F | GAGGAAGATCACAAGTTCGATTTCTGAGCTAGCAATTTCCCCGATCGTTCAAAC | To construct pCB301-2μ-L-opt |
| pCB/L-opt/R | TTGTTGTTGGTAATTGTTGTAAAAATACCATGGCCTCTCCAAATGAAATGAACTTCC |  |
| L-opt/del DD/F | AACATGGAGTCATCTTCATCATTCATCATCTCTATCCCA | To construct pCB301-2μ-L-opt^dDD^ |
| L-opt/del DD/R | GATGATGAATGATGAAGATGACTCCATGTTGTTC |  |
| vRNA1/pCB/F | TTTCATTTGGAGAGGACACAAAGTCCAGAGGAAAACA | To construct pCB301-2μ-vRNA1 |
| vRNA1/pCB/R | ATGCCATGCCGACCCACACATAGTCAGAGGAAAAAATAATTTTG |  |
| GFP/R | TTACTTGTACAGCTCGTCCATG | To construct the vRNA1 MR_GFP_ plasmid |
| GFP/F | ATGGTGAGCAAGGGCGAG |  |
| vRNA1/backbone/F | GCCCTTGCTCACCATCCTTCAATTCTTTTGTGGAAAAC |  |
| vRNA1/backbone/R | GAGCTGTACAAGTAAGAAGTCCTCTCCAACAAAGGA |  |
| vRNA3/HHRzpart/F | AAGGCCGAAACTATAGGAATATCTTCCTATAGTCACACAAAGTCCTGGGTAAAATAG | To construct the pCB301-vRNA3 plasmid |
| vRNA3/pCB/R | ATGCCATGCCGACCCACACAAAGTCTGGGTAATAAAATTTTC |  |
| 35S/HHRz/R | TATAGTTTCGGCCTTTCGGCCTCATCAGACACAAAGAGGCCTCTCCAAATGAAATG |  |
| HDV/F | GGGTCGGCATGGCATCTCCAC |  |
| RFP/F | ATGGCCTCCTCCGAGAACG | To construct the vRNA3 MR_RFP-GFP_ plasmid |
| RFP/R | TTACAGGAACAGGTGGTGGCG |  |
| RNA3/IGR/F | CACCTGTTCCTGTAAAAATAAAAAGAAAAAAGAAAAAAGAAAATAAAAC |  |
| RNA3/IGR/R | GAGCTGTACAAGTAAACTGAACAAGTCAGTAGTTG |  |
| vRNA3/backbone/F | GCCCTTGCTCACCATTGTAGCAAGAGGTACTGGAG |  |
| vRNA3/backbone/R | CTCGGAGGAGGCCATACTTAGATGATGTCGGAATTG |  |
| vRNA2/HDV/R | ATGCCATGCCGACCCGGGACACAAAGTCTGGGTATAACTTC | To construct the pCB301-vRNA2 plasmid |
| vRNA2/HHRz/F | GGAATTCCTATAGTCACACAAAGTCCTGGGTATATAAG |  |
| HHRz//backbone/R | GACTATAGGAATTCCTTTCCTATAGTTTCGGC |  |
| HDV/F | GGGTCGGCATGGCATCTCCAC |  |
| vRNA2/backbone/R | CTCGGAGGAGGCCATTGCTGAAGACGAGATTCTCCG | To construct the vRNA2_RFP-GFP_ plasmid |
| vRNA2/backbone/F | GCCCTTGCTCACCATATTAAAGATTGTGGTGTAGATGAAATATG |  |
| RNA2 IGR/F | CACCTGTTCCTGTAAACTTGGGAGATGATATTGACTG |  |
| RNA2 IGR/R | GAGCTGTACAAGTAAGCCAATAGGTTCACTCATGTATG |  |
| vRNA2/pCB/F | TTTCATTTGGAGAGGACACAAAGTCCTGGGTATATAAGC | To construct the vRNA2 MR_NS2-GFP_ plasmid |
| vRNA2/HDV/R | ATGCCATGCCGACCCGGGACACAAAGTCTGGGTATAACTTC |  |
| RNA3/del 5'UTR/F | GGAATTCCTATAGTCATGGCCTCCTCCGAGAAC | To delete the 5' UTR of vRNA3 MR_RFP-GFP_ |
| vRNA3/pCB/R | ATGCCATGCCGACCCACACAAAGTCTGGGTAATAAAATTTTC |  |
| GFP/RFP/R | CACCTGTTCCTGTAATTACTTGTACAGCTCGTCCATG | To delete the IGR3 of vRNA3 MR_RFP-GFP_ |
| vRNA3/pCB/R | ATGCCATGCCGACCCACACAAAGTCTGGGTAATAAAATTTTC |  |
| vRNA3/HHRzpart/F | AAGGCCGAAACTATAGGAATATCTTCCTATAGTCACACAAAGTCCTGGGTAAAATAG |  |
| RFP/R | TTACAGGAACAGGTGGTGGCG |  |
| RNA3/del 3'UTR/R | ATGCCATGCCGACCCATGGTGAGCAAGGGCGAG | To delete the 3' UTR of vRNA3 MR_RFP-GFP_ |
| vRNA3/HHRzpart/F | AAGGCCGAAACTATAGGAATATCTTCCTATAGTCACACAAAGTCCTGGGTAAAATAG |  |
| cRNA1/pCB/F | TTTCATTTGGAGAGGACACATAGTCAGAGGAAAAAATAATTTTG | To construct the pCB301-cRNA1 plasmid |
| cRNA1/pCB/R | ATGCCATGCCGACCCACACAAAGTCCAGAGGAAAACA |  |
| cRNA1/backbone/F | GAAGTGGAATAACCAAAGGTGGTGTAGTCATCCTTCAATTCTTTTGTGGAAAAC | To construct the cRNA1_L-opt_ plasmid |
| cRNA1/backbone/R | CAAGAGGAAGATCACAAGTTCGATTTcTGAGAAGTCCTCTCCAACAAAGGAAC |  |
| cRNA3/pCB/F | TTTCATTTGGAGAGGACACAAAGTCTGGGTAATAAAATTTTCGATTTTG | To construct the cRNA3 MR_N-RFP_ plasmid |
| RNA3/IGR/F | CACCTGTTCCTGTAAAAATAAAAAGAAAAAAGAAAAAAGAAAATAAAAC |  |
| RFP/R | TTACAGGAACAGGTGGTGGCG |  |
| cRNA3/pCB/R | ATGCCATGCCGACCCACACAAAGTCCTGGGTAAAATAGTTATATTTTTAC |  |
| cRNA4/pCB/F | TTTCATTTGGAGAGGACACAAAGTCAGGGCATATCTTTTG | To construct the pCB301-cRNA4 plasmid |
| cRNA4/pCB/R | ATGCCATGCCGACCCACACAAAGTCCAGGGCATTTG |  |
| cRNA4/backbone/F | CTCGGAGGAGGCCATCTTCGATTCTGATTAAATTGCTG | To construct the cRNA4 MR_MP-RFP_ plasmid |
| cRNA4/backbone/R | CCACCTGTTCCTGTAATTTATTGCATATCACATTTTCCACC |  |
| RFP/F | ATGGCCTCCTCCGAGAACG |  |
| RFP/R | TTACAGGAACAGGTGGTGGCG |  |
